# Acceptability of a Mobile Phone App for Measuring Time Use in Breast Cancer Survivors (Life in a Day): Mixed-Methods Study

**DOI:** 10.2196/cancer.8951

**Published:** 2018-05-14

**Authors:** Matthew Cole Ainsworth, Dori Pekmezi, Heather Bowles, Diane Ehlers, Edward McAuley, Kerry S Courneya, Laura Q Rogers

**Affiliations:** ^1^ Department of Health Behavior University of Alabama at Birmingham Birmingham, AL United States; ^2^ National Cancer Institute Division of Cancer Prevention Bethesda, MD United States; ^3^ Department of Kinesiology and Community Health University of Illinois at Urbana-Champaign Urbana, IL United States; ^4^ Faculty of Physical Education and Recreation University of Alberta Edmonton, AB Canada; ^5^ Department of Nutrition Sciences University of Alabama at Birmingham Birmingham, AL United States

**Keywords:** cancer, technology, time management, mHealth, physical activity

## Abstract

**Background:**

Advancements in mobile technology allow innovative data collection techniques such as measuring time use (ie, how individuals structure their time) for the purpose of improving health behavior change interventions.

**Objective:**

The aim of this study was to examine the acceptability of a 5-day trial of the Life in a Day mobile phone app measuring time use in breast cancer survivors to advance technology-based measurement of time use.

**Methods:**

Acceptability data were collected from participants (N=40; 100% response rate) using a self-administered survey after 5 days of Life in a Day use.

**Results:**

Overall, participants had a mean age of 55 years (SD 8) and completed 16 years of school (SD 2). Participants generally agreed that learning to use Life in a Day was easy (83%, 33/40) and would prefer to log activities using Life in a Day over paper-and-pencil diary (73%, 29/40). A slight majority felt that completing Life in a Day for 5 consecutive days was not too much (60%, 24/40) or overly time-consuming (68%, 27/40). Life in a Day was rated as easy to read (88%, 35/40) and navigate (70%, 32/40). Participants also agreed that it was easy to log activities using the activity timer at the start and end of an activity (90%, 35/39). Only 13% (5/40) downloaded the app on their personal phone, whereas 63% (19/30) of the remaining participants would have preferred to use their personal phone. Overall, 77% (30/39) of participants felt that the Life in a Day app was *good* or *very good*. Those who agreed that it was easy to edit activities were significantly more likely to be younger when compared with those who disagreed (mean 53 vs 58 years, *P*=.04). Similarly, those who agreed that it was easy to remember to log activities were more likely to be younger (mean 52 vs 60 years, *P<*.001). Qualitative coding of 2 open-ended survey items yielded 3 common themes for Life in a Day improvement (ie, convenience, user interface, and reminders).

**Conclusions:**

A mobile phone app is an acceptable time-use measurement modality. Improving convenience, user interface, and memory prompts while addressing the needs of older participants is needed to enhance app utility.

**Trial Registration:**

ClinicalTrials.gov NCT00929617; https://clinicaltrials.gov/ct2/show/NCT00929617 (Archived by WebCite at http://www.webcitation.org/6z2bZ4P7X)

## Introduction

### Background

As mobile phone technology becomes more widely accessible, so does its potential to act as a platform for high-reach physical activity promotion with increased personalization. This area of research is particularly relevant for breast cancer survivors, as it remains one of the most common cancers among women, regardless of race or ethnicity, with approximately 252,710 expected new cases in 2017 [1]. Moreover, it has been recently estimated that over 3.1 million US women either have a history of breast cancer or have a current cancer diagnosis [2]. Interventions targeting physical activity are common, as it is one of the few modifiable risk factors for breast cancer development and outcomes [3]. However, a majority of breast cancer survivors fail to achieve the US Department of Health and Human Services federal guidelines of 150 min per week of moderate intensity physical activity [4]. This is of particular concern as inactivity and sedentary behaviors have been shown to be a risk factor independent of physical activity [5,6]. Furthermore, recent emphasis has been placed on the importance of promoting leisure-time physical activity for mortality benefits [7-9]. To address these high rates of physical inactivity, effective interventions are needed. A better understanding of activity patterns and time use among survivors would help inform these efforts by providing a more comprehensive evaluation of an individual’s day-to-day activities.

### Gaps in the Literature

One limitation of physical activity research to date has been inadequate data relevant to the activitystat hypothesis, which suggests that an increase in physical activity in 1 domain often leads to a decrease in another domain in an effort to keep energy expenditure constant through biological regulation [10,11]. Moreover, recent research aimed at examining shifts in time-use domains found that domains such as Physical Activity, Self-Care, and Active Transport increased, whereas Television/Videogames domains decreased after a structured exercise intervention [12,13]. Failure to recognize shifts in activity domains could lead to inaccurate postintervention assessments of physical activity and time-use measurements, which have been shown to be important tools for elucidating the actual impact of physical activity program [14]. Furthermore, it is theorized that self-awareness can be promoted by bringing attention to one’s behavior in close temporal proximity to its occurrence, which may influence behavioral and cognitive changes [15]. Therefore, technology-supported time-use measurements may be advantageous for both physical activity measurement and promotion.

In general, many published studies in this area of research have utilized the Multimedia Activity Recall for Children and Adolescents (MARCA), a computerized self-report instrument for time-use measurement [14], which has since been adapted for use among adult populations and demonstrated both validity and reliability [16]. The MARCA has also been applied in a variety of settings, and previous uses include examining activity patterns among older Australian workers [17] and adolescents [18-20], as well as 5-year-old children [21]. Despite wide applicability of the MARCA, one limitation of currently available measurements include inability to provide a continuous measurement of time use, as it relies on 24-hour recall rather than real-time assessment within the context of daily life. Moreover, a mobile phone version of the MARCA does not currently exist, which limits its applicability in an increasingly wireless environment. In an effort to address this, this study utilized a time-use measurement app named Life in a Day that allows participants to track activities throughout their day. Life in a Day is a mobile app that was developed by the Division of Cancer Control and Population Sciences at the US National Cancer Institute in collaboration with MEI Research, Ltd. The app allows self-tracking of customizable activities (eg, personal care, house cleaning, walking the dog), which offers researchers insight into how people utilize their time. To our knowledge, no other study has examined time use among breast cancer survivors. Data regarding the acceptability of such a measure is critical to further research testing how time-use alterations could be employed to optimize physical activity promotion in this at-risk population. The purpose of this study was to evaluate the acceptability of the Life in a Day app for time use among breast cancer survivors recruited from 2 (one Midwestern and one Southeastern) US cities. Moreover, this study explored the relationship between baseline characteristics of participants and Life in a Day user experiences.

## Methods

### Study Design

This study utilized a posttest-only, embedded evaluation research design with concurrent quantitative and qualitative data collection [22]. Self-administered participant satisfaction surveys were completed after a 5-day trial of a time-use measurement app by a subsample of breast cancer survivors completing baseline assessments in a larger randomized physical activity-controlled trial (registered on ClinicalTrials.gov, NCT00929617). Approval for this study was granted by the Institutional Review Boards at both participating study sites, and informed consent was obtained before initiating study activities.

### Participants

Participants in this study included adult women aged 18 to 70 years with a history of ductal carcinoma in situ or stage I-IIIA breast cancer who had completed primary treatment (ie, surgery, radiation, and/or chemotherapy). All participants met eligibility criteria for the parent study, which are described in detail in a previous report [23] and included being ≥8-weeks post surgery, English speaking, medically cleared by a physician, and insufficiently active (ie, ≤30 min of vigorous physical activity or ≤60 min of moderate physical activity per week, on average, during the past 6 months). Exclusion criteria for the larger parent study also included the following: (1) dementia or organic brain syndrome; (2) medical, psychological, or social characteristics that would interfere with ability to fully participate in program activities and assessments (eg, psychosis and schizophrenia); (3) contraindication to participation in a regular physical activity program; (4) metastatic or recurrent disease; (5) inability to ambulate; and (6) elective surgery planned during the duration of the intervention, which would interfere with intervention participation (eg, breast reconstructive surgery). As previously described [23], strategies for recruitment included community advertising, worksite email lists, and medical network channels (eg, physician referrals).

### Protocol

**Figure 1:**
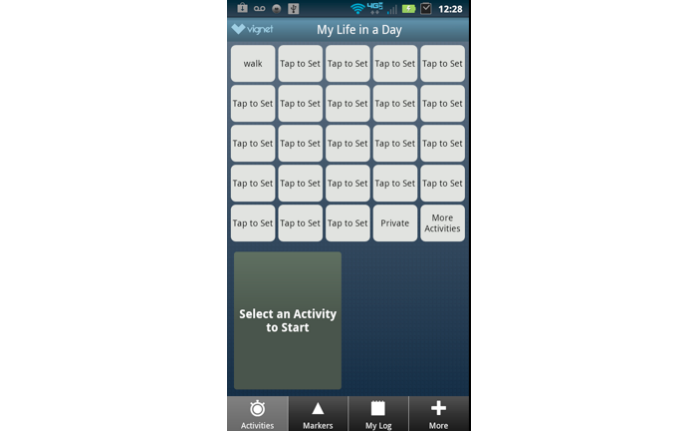
Customizable activity buttons for Life in a Day time use mobile app.

Following study enrollment, participants attended an orientation session in which they either received a mobile device (ie, Android) with the Life in a Day time-use measurement app (National Cancer Institute prototype version) installed or chose to download the app on their personal phone if it was an Android device. Staff instructed participants on how to generate a user profile and log daily activities within the app. Participants also received a paper-based start-up instruction guide with this information for reference if needed. Participants had access to 23 user customizable activity buttons, one private button, and a *more activities* button (see Figure 1). When customizing activity buttons, the search term was queried against a keywords list and matching activity descriptions were displayed. If no suitable activity description was listed, participants could then create a new activity. Other features of Life in a Day included a start and stop timer for tracking, the option to track concurrent activities, and the ability to edit logged activities.

**Figure 2:**
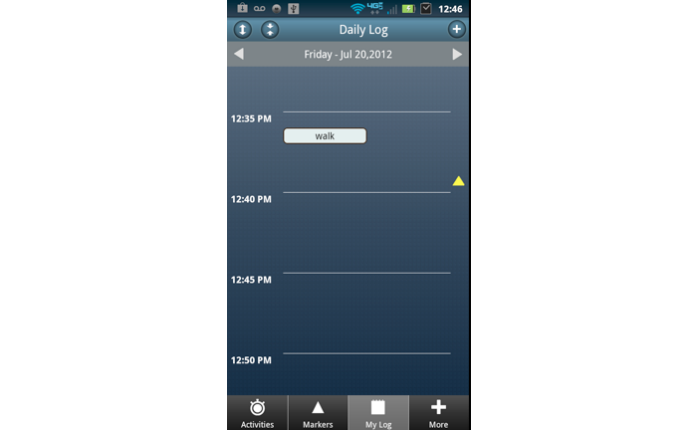
Daily log with example activity for Life in a Day time use mobile app.

Following orientation, participants were asked to use the time use app to log all activities for 5 consecutive 24-hour days (including sleep time) by pressing the appropriate customized button at the beginning of the activity and again at the end. When logging activities during this period, participants were asked to select up to 3 categories (eg, walking, errand, or appointment) to identify the purpose of the activity. Participants could review and, if necessary, edit tracked activities from the daily log screen of the app (see Figure 2). For the purpose of this study, activity was not limited to physical activity. After the 5-day trial was completed, research staff members double-checked the phone to ensure all time was tracked. Participants then returned the study-provided mobile device to study staff and completed a questionnaire assessing functionality and satisfaction with the time use app (see Multimedia Appendix 1).

### Measurements

#### Demographic and Memory Measures

A self-administered questionnaire assessed baseline demographics (age, race or ethnicity, education, household income, and marital status). Participants also self-reported cancer-related information such as cancer stage, treatment(s) received, and time since treatment. As memory could influence acceptability of the app use, participants self-reported memory difficulties using the 10-item Frequency of Forgetting scale using a 7-point Likert scale [24]. Higher scores indicate less perceived memory difficulty. The subscore used in this study had a possible range of 5-35 (based on 5 of the 10 items). This scale has previously demonstrated reliability and construct validity similar to the respective 33-item version [24].

#### Life in a Day Satisfaction Questionnaire

A self-administered 16-item questionnaire assessed functionality and participant experiences with the time use app (see Multimedia Appendix 1). The questionnaire was designed based upon principles of technology adoption [25,26] and included a mixture of 5-point Likert-scale, yes or no, and open-ended items on various qualities of the app. Participants were asked to rate their agreement with statements such as “Learning to use the Life in a Day app was easy,” “Navigating the Life in a Day app was clear and understandable,” and “I enjoyed using the Life in a Day app.” Likert-scale items ranged from 1 (Completely Disagree) to 5 (Completely Agree). Additionally, participants were asked to rate the Life in a Day app for tracking activity on a scale of 1 (Very Poor) to 5 (Very Good). Participants were also asked if they downloaded the app on their personal phone. One open-ended item asked participants who did not agree that “it was easy to remember to log their activities using the app” to suggest what could be done to make it easier. This item was limited to those who disagreed with the statement to minimize unnecessary participant burden. Another open-ended item asked all participants to provide the research team with any other comments on the Life in a Day app. Open-ended items were independently coded by 3 research team members using a conventional content analysis approach, in which codes are derived from the data and defined during qualitative data analysis [27,28]. The coders compared passages, resolved discrepancies in the coding, and agreed on the coding for each evaluation response. Themes from the feedback emerged and are described below.

### Statistical Analysis

Statistical analyses were conducted using SAS 9.4 (SAS Institute Inc, USA). Sample characteristics and Life in a Day satisfaction questionnaire data were summarized using descriptive statistics. Independent samples *t*-tests were conducted to examine the relationship between sample characteristics and satisfaction questionnaire responses. Survey items using a 5-point Likert-scale were categorized as either disagree (score of 1-3) or agree (score of 4 or 5) to assess potential associations between sample characteristics and agreement status for each questionnaire item.

## Results

### Participant Characteristics

A total of 40 participants (response rate of 100%) completed the satisfaction questionnaire after a 5-day trial of the Life in a Day mobile phone app. Sociodemographic, cancer-related, and self-reported memory characteristics are presented in Table 1.

**Table 1:** Baseline sociodemographic, cancer, and memory characteristics (N=40).

Characteristics		Statistics
Gender (female), n (%)		40 (100)
Age in years, mean (SD)		55 (8)
Education in years, mean (SD)		16 (2)
**Race/ethnicity, n (%)**		
	White	29 (73)
	African American	9 (22)
	Other	2 (5)
**Annual household income (US $), n (%)^a^**		
	<10,000	2 (5)
	10,000-19,999	1 (3)
	20,000-34,999	4 (10)
	35,000-49,000	6 (15)
	≥50,000	26 (65)
**Marital status, n (%)**		
	Single	3 (8)
	Married	22 (55)
	Divorced/separated	9 (22)
	Widowed	4 (10)
	Not married	2 (5)
**Cancer stage, n (%)**		
	0	4 (10)
	I	17 (42)
	II	15 (38)
	III	4 (10)
	IV	0 (0)
**Prior chemotherapy treatment, n (%)**		
	Yes	30 (75)
	No	10 (25)
**Prior radiation treatment, n (%)**		
	Yes	22 (55)
	No	18 (45)
**Time since diagnosis, n (%)**		
	Less than 1 year	4 (10)
	1 to <2 years	12 (30)
	2 to <3 years	7 (18)
	3 to <4 years	6 (15)
	4 to <5 years	2 (5)
	5 or more years	9 (22)
Frequency of forgetting (subscore; possible range 5-35)		23 (6)

^a^n=39.

Overall, participants had a mean age of 55 years (SD 8) and completed 16 years of school (SD 2). Moreover, the study sample was a majority white (73%, 29/40), married (55%, 22/40), and had an annual income ≥US $50,000 (65%, 26/39). All participants enrolled in the study were female, and most had undergone prior chemotherapy (75%, 30/40) or radiation (55%, 22/40) treatments. Reported time since cancer diagnosis varied between participants, although most (90%, 36/40) indicated that it had been more than a year.

### Acceptability of Life in a Day

A summary of quantitative responses to Life in a Day evaluation questionnaire items is presented in Multimedia Appendix 2. Participants generally agreed that the time use app was easy to learn (83%, 33/40) and would prefer to use it compared with paper-and-pencil activity tracking (73%, 29/40). Furthermore, 60% (24/40) of participants felt that neither did they find tracking their time use with the app for 5 days as too much nor was it too time-consuming (68%, 27/40). Most agreed that the app was easy to read (88%, 35/40) and navigate (80%, 32/40) on the mobile phone and that it was easy to log activities using the activity timer (90%, 35/40). Overall, 77% (30/40) of participants rated the Life in a Day app as good or very good.

**Figure 3:**
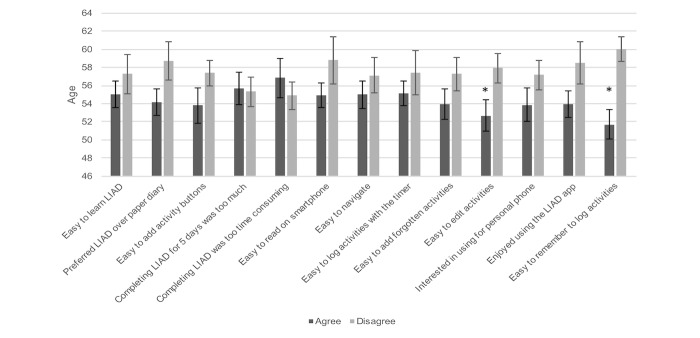
Life in a Day participant mean age by survey item agreement. All statistically significant (P<.05) interactions are denoted with an asterisk (*).

Participant age was found to be associated with 2 Life in a Day survey items. Participants who agreed it was easy to edit activities were statistically significantly younger when compared with those who disagreed (mean 53 vs 58 years, *P*=.04). Similarly, those who agreed that it was easy to remember to log activities were more likely to be younger than those who disagreed (mean 52 vs 60 years, *P*<.001). Figure 3 displays the mean age by agreement status for each questionnaire item. Educational attainment, frequency of forgetting, and study site were not associated with survey responses.

### Qualitative Feedback Related to Life in a Day and Suggestions for App Improvements

The qualitative dataset consisted of 35 comments across 2 survey items from the sample of 40 participants. For survey item 13a (“what could have made it easier?”), 14 out of 40 (35%) participants provided responses. Moreover, 21 out of 40 (53%) participants provided responses to survey item 16 (other comments). A total of 26 out of 40 participants (65%) responded to at least one of the open-ended survey items, whereas 9 provided comments on both. Qualitative coding of these 2 open-ended survey items yielded several major themes for improving the Life in a Day app. A list of themes and subthemes identified via conventional content analysis is provided in Textbox 1.

#### Participant Feedback Related to Ease of Remembering to Log Activities in the Time Use App for Cancer Survivors

Participants only completed this open-ended follow-up item if they stated that it was not easy to remember to log activities with the app. A total of 14 participants completed this open-ended item. As noted in Figure 1, this item was significantly associated with participant age. Participants who completed this open-ended item had a mean age of 60 years, which was slightly higher than that of the sample. A mixed-methods data joint display of participant feedback (ie, representative quotes) by age category (<60 years vs ≥60 years) is presented in Table 2. Age categories were determined by the mean age of respondents and are provided to allow for the comparison of perspectives from younger and older participants.

One theme that emerged from the responses was the need for an adjustment period when first using the app with practice time being particularly important for the participants ≥60 years. Issues with multitasking were commonly identified in participant responses (both age groups). Difficulties arose with activity tracking when participants were involved in activities such as running errands or caretaking. In addition to difficulties with tracking activity due to multitasking, some participants expressed the need for a built-in reminder system for the app, with older participants expressing difficulty with the cognitive load burden specifically (Table 2). Finally, older participants indicated difficulty with the app interface due to unfamiliarity with technology.

**Themes and subthemes from open-ended survey items. Item 13a “What could have made it easier?” was answered by 14 participants and item 16 “Other comments” was answered by 21 participants.**

**Item 13a. What could have made it easier?**

- User interface
  Multitasking
- Convenience
  - General
  - Personal phone
  - Wearability
- Reminders
- Adjustment period
- Improved set-up/orientation
- Platform expansion
- Minimization of cognitive overload

**Item 16. Other comments**

- User interface
  - Adding activities
  - Changing activities
  - Changing categories
  - Choosing categories
  - Editing activities
  - Multitasking
- Convenience
  - Personal phone
  - Readability
  - Wearability
- Burden
  - General
  - Easier than pen and paper
  - Preferred pen and paper
  - Time
- Adjustment period
- Improved training
- Insight into time use
- Instruction clarity
- Minimization of cognitive overload
- Comfort with technology

**Table 2:** Joint display of participant responses by age category.

Item			Representative open-ended responses
**What could have made it easier?^a^**			
	**Age <60 years**		
		Participant A	“Trial was too short. I needed a small period to get adjusted to having the app.”
		Participant B	“If there could be a way to have this device on you, it would be easier to remember to change the activities. Often I had to go find where my phone was, and if I could not locate it, I could not ‘call it’ to locate it!”
		Participant C	“I didn’t add enough activities to cover my day adequately and did not adjust it once I left the office.”
		Participant D	“...give me a electric shock so that I would remember.”
	**Age ≥60 years**		
		Participant E	“My biggest problem was remembering to change from one activity to another—running errands was a real pain!”
		Participant F	“I did not have a place to keep it. I had to wear pockets and it was difficult to remember to carry it. Would be focused on other things.”
		Participant G	“It is hard to remember to track every activity. A sound prompt every 1/2 to 2 hours to remind you to check and see if you are on track and logging the correct or current activity. For example, when you are on the go, and not thinking ahead from 1 thing to the next.”
		Participant H	“...time/practice [this was all new so it was easy to forget].”
**Other comments^b^ you have regarding the Life in a Day app**			
	**Age <60 years**		
		Participant B	“I found it awkward keeping up with my phone; my real cell phone; the paper handout describing the quick start guide, especially if I was multitasking. Much of my 5 days usage was with my mom who is in a wheelchair, is diabetic, and requires much help. So as I said, trying to keep up with her, keep up with the phone, change my activities, go back to her, take care of myself and family and things I needed to do, go find the phone to change my activities, etc. did become somewhat overwhelming and confusing. Perhaps a device that can be on the person and simplified would be better [at least for people like me!]”
		Participant I	“…I found it interesting to document my day. Hopefully, it will encourage me to make some changes for the good to my lifestyle.”
		Participant J	“I’m not particularly savvy with the use of all smart ph. I have a blackberry. Honestly, I probably needed a bit more training but my fault for not asking.”
	**Age ≥60 years**		
		Participant K	“It would be easy to track if you did the same activity for 6-8 hours. However, I might sit down and do accounting for my company, then jump up and load clothes, then jump in the car and travel to the store. I have too many activities during the day for this app. I felt as if it ‘took over my life.’ Not good for an active person that changes activities all day long.”
		Participant F	“Did not do correct categories. Item was easy, I was the problem. Does not come easily for me so when I am focused on doing my responsibilities using app suffered.”
		Participant H	“I would have less of a problem if this was not a brand new thing for me. The phone seemed to have a mind of its own sometimes. It did not function as easily as it should have probably because I didn't know how to correct an error or find the right item when it went astray.”
		Participant E	“I felt the activity tracker did not provide a way to accurately track my activity. TV time, for example, does not mean long term activity as I am constantly up- getting dogs in and out, taking care of my husband, answering the phone, etc.”

^a^Representative open-ended responses chosen from the 14 respondents.

^b^Representative open-ended responses chosen from the 21 respondents.

#### Participant Feedback Related to General Comments About the Time Use App

Participants were asked if they had any comment regarding the app, and these responses also highlighted issues regarding comfort with technology and burden (eg, time). A total of 21 participants completed this open-ended item. Several participants described experiences in which it was difficult or inconvenient to operate the app due to it not being installed on a personal phone or available in a platform for wearable devices. As presented in Table 2, one participant explained how these limitations made it inconvenient for tracking activity.

Some participants identified barriers to activity tracking as it relates to the Life in a Day app user interface. Specifically, scenarios involving numerous successive activities were often referenced, and participants found it difficult to perform tasks such as adding or editing activities in these situations. As mentioned before, quantitative data from the survey indicates that older cancer survivors were significantly less likely to agree that it was easy to edit activities. Participants highlighted the need for a more user-friendly interface for individuals with busy lifestyles. Despite the aforementioned limitations with the Life in a Day trial, participants did anticipate positive benefits from utilizing the Life in a Day app.

## Discussion

### Principal Findings

This mixed-methods study of the Life in a Day time use app provides insight into the acceptability of utilizing mobile apps for activity tracking among breast cancer survivors and advances efforts to address physical inactivity among this population. The Life in a Day app for time-use measurement demonstrated satisfactory acceptability (ie, favorable satisfaction questionnaire ratings), with 77% (30/39) rating it as *Good* or *Very Good*. From our qualitative examination of responses to the satisfaction survey, several themes were identified. Although participants indicated overall satisfaction with the time use app, events involving multitasking or consecutive activities were often portrayed as a barrier to successful activity tracking, and participants made suggestions for helping them remember to change activities within the app (ie, sound prompts). An additional barrier to tracking included the burden of carrying an extra phone due to limited platform availability (ie, Android devices only at time of the study). This was especially relevant in situations involving aforementioned multitasking, and participants suggested the incorporation of reminder prompts or wearable devices might help alleviate difficulties with tracking in these scenarios. Differences in quantitative responses by older participants may have been related to difficulties expressed with the cognitive load burden and app interface.

Our utilization of quantitative data allowed further exploration into characteristics (ie., age, education, frequency of forgetting) that may have contributed to satisfaction survey responses. Although no associations were found regarding education or frequency of forgetting, results from the analyses indicated that age was significantly associated with both perceived ease of editing activities and ease of remembering to log activities. These findings suggest that older cancer survivors may have increased difficulty when engaged in these 2 aspects of mobile activity tracking. Responses to several other elements of Life in a Day were found to have an agreement rate >80% (ie, easy learning to use the app, easy to read, clear navigation, easy to log activities). These responses were not significantly associated with age and highlight strengths of the app perceived by the overall sample rather than younger or older cancer survivors only. Recently, a 2016 study of health intervention delivery modalities among cancer survivors found that age was negatively correlated with preferences for mobile phone apps [29]. However, results from this study suggest that modality preferences may be shifting, particularly among female breast cancer survivors.

To our knowledge, this is the first trial testing the acceptability of a time use app in cancer survivors. Additionally, Life in a Day goes beyond existing time use-measurement tools such as the previously described computerized MARCA by utilizing a platform for select mobile devices.

Life in a Day also aims to address limitations associated with 24-hour recall by creating opportunities for real-time assessment, although the option for recall assessment could be used if an activity was missed.

However, some relevant ecological momentary assessment (EMA) studies have been conducted. Although EMA can be used to measure time use, it is distinct from the current app in that EMA uses repeated sampling techniques (eg, every 45 min) to measure behavior or experience rather than continuous, ongoing measurement and, thus, may rely more on retrospection. Moreover, EMA is generally used to study specific behaviors of interest (eg, panic attack/s) [30] compared with general time use activities as in this study and gives the Life in a Day app more general, widespread application for assessing lifestyle behaviors.

Moreover, 2 past EMA studies were conducted on specific behaviors (vs general time use) in populations similar to this study (eg, sleep, symptoms, and mood among breast cancer patients receiving chemotherapy [31] and exercise adoption among endometrial cancer survivors [32]). These studies involved longer-term assessments (3 daily assessments for 3 weeks and twice daily assessments for 10- to 12-day periods every 2 months for a total of 6 months, respectively) than this study (5 consecutive days); however, the data collection relied more on retrospection (vs real-time assessment) and occurred via handheld computers (vs mobile phone).

Another 2 EMA studies were conducted in a different population (college students), with 1 work focused on mind wandering [33] and the other on general time use (vs specific behavior/s), like this study [34]. These examinations differed from the prior 2 EMA studies as assessments were conducted via an app on mobile phones/PDAs designed to capture activities in the past hour or 20 min, respectively (involved less retrospection). Comparisons with this study include similar or longer follow-up periods (hourly assessments for 1 week and twice daily assessments for 3 weeks, respectively) and the use of text messages [33] or push notifications with alarms [34] to prompt participant responses. Our study required participant initiation of the app to track time use. Given that some participants in this study requested reminder prompts, however, incorporating this as an optional function could benefit future studies exploring time use among cancer survivors.

### Strengths and Limitations

Overall strengths of the trial include the use of innovative technology to provide insights into time use of cancer survivors with generally high rates of physical inactivity. Additionally, our mixed-methods approach allowed for a more in-depth understanding of participant experiences with the Life in a Day app. Moreover, our data can assist with developing interventions to improve acceptability and use among older individuals. Limitations of this study include the use of open-ended questions that may limit the breadth of qualitative data obtained as exemplified by the small number of responses across our 2 open-ended survey items. Nevertheless, data obtained yielded qualitative information that expanded our understanding of the age differences noted, with the quantitative data achieving the purpose desired when using a mixed-methods approach. Our study was also limited by a small sample size and completion of the evaluation after only 5 days of app use. Moreover, limited platform availability at the time of the study (Android phones only) may have restricted the number of participants who downloaded the app on their personal phone, which may limit generalizability to future use as platform availability increases. For those participants unable to download the app, the adoption of an extra phone might have confounded acceptability findings. Furthermore, time spent orienting participants to the app might be considered a limitation of this study, as participants attended one 30-min session before beginning the trial to learn the Life in a Day time-use system. Our findings also might not be generalizable beyond groups meeting the study inclusion criteria (eg, noncancer survivors).

### Conclusions and Implications

This line of research explores the acceptability of mobile time-use measurement among breast cancer survivors and has potential for informing future physical activity intervention development. Although further study is needed to determine usability of the Life in a Day time use app, this study demonstrated acceptability among this population, with survey responses highlighting areas of improvement in which future research should address. Our quantitative analyses indicate that participants generally perceived adding forgotten activities in the app as difficult, regardless of age. This finding suggests an area of improvement relevant to all survivors. This study also has several public health implications. First, such apps require further refinement and testing but will likely provide more accurate time-use data than retrospective surveys and can be used to augment documentation of physical activity recorded by accelerometry. Additionally, such apps could help promote better health in cancer survivors by making them more aware of their habits and providing potential insights into how and when physical activity could be added to their daily life. The integration of such apps could substantially benefit public health, given the rising number of survivors and the large need for physical activity in this population.

## Appendix

Multimedia Appendix 1 Life in a Day phone app evaluation.

Multimedia Appendix 2 Life in a Day survey item and percent agreement (N=40).

## Abbreviations

EMA: ecological momentary assessment
MARCA: Multimedia Activity Recall for Children and Adolescents
