# Supplementary material for: Acceptability of a Mobile Phone App for Measuring Time Use in Breast Cancer Survivors (Life in a Day): Mixed-Methods Study
Source: JMIR Cancer. 2018 May 14;4(1):e9. doi: 10.2196/cancer.8951 (PMC5972204; doi:10.2196/cancer.8951)
Supplement: Multimedia Appendix 1 [file cancer_v4i1e9_app1.pdf]

# Life In A Day (LIAD) Phone App EVALUATION

**Instructions:** Please rate your agreement with the following statements on a scale of 1 to 5, with 1 meaning that you "completely disagree" and 5 meaning that you "completely agree".

**1-Completely disagree** **2 -Somewhat Disagree** **3-Neither Agree nor Disagree** **4 -Somewhat Agree** **5-**Completely agree

| | | | | | |
|---|---|---|---|---|---|
| 1. Learning to use the LIAD app was easy | 1 | 2 | 3 | 4 | 5 |
| 2. I would prefer to log activities using the electronic LIAD app over using a paper & pencil diary | 1 | 2 | 3 | 4 | 5 |
| 3. It was easy to add new activity buttons to the activity screen | 1 | 2 | 3 | 4 | 5 |
| 4. Completing the LIAD app for 5 consecutive days was too much | 1 | 2 | 3 | 4 | 5 |
| 5. Completing the LIAD app was too time consuming each day | 1 | 2 | 3 | 4 | 5 |
| 6. The LIAD app was easy to read on the smartphone | 1 | 2 | 3 | 4 | 5 |
| 7. Navigating the LIAD app was clear and understandable | 1 | 2 | 3 | 4 | 5 |
| 8. It was easy to log activities by pressing the activity timer at the start and end of an activity | 1 | 2 | 3 | 4 | 5 |
| 9. It was easy to add activities if I forgot to log them at the time of the activity | 1 | 2 | 3 | 4 | 5 |
| 10. It was easy to go back and edit activities if necessary | 1 | 2 | 3 | 4 | 5 |
| 11. I would be interested in using the LIAD app for my personal use | 1 | 2 | 3 | 4 | 5 |
| 12. I enjoyed using the LIAD app | 1 | 2 | 3 | 4 | 5 |
| 13. It was easy to remember to log my activity in the LIAD app<br>*a. If you disagree, what could have made it easier?* | 1 | 2 | 3 | 4 | 5 |

16. Please provide an overall rating of the LIAD app for tracking your activity

| **Very Poor** | **Poor** | **Fair** | **Good** | **Very Good** |
|---|---|---|---|---|
| 1 | 2 | 3 | 4 | 5 |

| | | |
|---|---|---|
| 17. Did you download the app on your personal phone? | Yes | No |
| *a. If no, would you have preferred to use your personal phone?* | Yes | No |

18. Please provide us with any other comments you have about the LIAD app below:
