# Supplementary material for: Acceptability of a Mobile Phone App for Measuring Time Use in Breast Cancer Survivors (Life in a Day): Mixed-Methods Study
Source: JMIR Cancer. 2018 May 14;4(1):e9. doi: 10.2196/cancer.8951 (PMC5972204; doi:10.2196/cancer.8951)
Supplement: Multimedia Appendix 2 [file cancer_v4i1e9_app2.pdf]

LiaD[a] survey items	Completely disagree, n (%)	Somewhat disagree, n (%)	Neither agree nor disagree, n (%)	Somewhat agree, n (%)	Completely agree, n (%)
Easy to learn LiaD	0 (0%)	3 (8%)	4 (10%)	8 (20%)	25 (63%)
Would prefer LiaD over paper diary	2 (5%)	5 (13%)	4 (10%)	5 (13%)	24 (60%)
Easy to add activity buttons	2 (5%)	3 (8%)	13 (33%)	8 (20%)	13 (33%)
Completing LiaD for 5 days was too much	15 (38%)	9 (23%)	4 (10%)	7 (18%)	5 (13%)
Completing LiaD was too time-consuming	19 (48%)	8 (20%)	2 (5%)	8 (20%)	3 (8%)
Easy to read on mobile phone	1 (3%)	2 (5%)	2 (5%)	7 (18%)	28 (70%)
Easy to navigate LiaD	1 (3%)	5 (13%)	2 (5%)	10 (25%)	22 (55%)
Easy to log activities with the timer (n=39)	0 (0%)	2 (5%)	2 (5%)	7 (18%)	28 (70%)
Easy to add forgotten activities	2 (5%)	7 (18%)	9 (23%)	13 (33%)	9 (23%)
Easy to edit activities	4 (10%)	6 (15%)	11 (28%)	12 (30%)	7 (18%)
Interested in	8 (20%)	8 (20%)	3 (8%)	11 (28%)	10 (25%)

using for personal phone					
Enjoyed using the LiaD app	2 (5%)	6 (15%)	5 (13%)	15 (38%)	12 (30%)
Easy to remember to log activities	5 (13%)	12 (30%)	1 (3%)	15 (38%)	7 (18%)
If you disagree, what could have made it easier? (n=14)	See text and Table 2				
	Very poor	Poor	Fair	Good	Very good
Overall rating of LiaD (n=39)	1 (3%)	1 (3%)	7 (18%)	17 (44%)	13 (33%)
	Yes	No			
Downloaded LiaD on personal phone	5 (12%)	35 (88%)			
If no, would you prefer to use personal phone (n=30)	19 (63%)	11 (37%)			
Other comments about LiaD (n=21)	See text and Table 2				

[a]LiaD: Life in a Day.
